# Magnetic Flux Density Measurement Platform for an Inductive Wireless Power Transmitter Coil Design

**DOI:** 10.3390/s22020479

**Published:** 2022-01-09

**Authors:** Nataša Prosen, Miro Milanovič, Jure Domajnko

**Affiliations:** Faculty of Electrical Engineering and Computer Science, University of Maribor, 2000 Maribor, Slovenia; miro.milanovic@um.si (M.M.); jure.domajnko2@um.si (J.D.)

**Keywords:** magnetic field, magnetic flux density, IPT, transmitter coil design, EM solver

## Abstract

This paper presents a platform developed for automated magnetic flux density measurement. The platform was designed to be used to measure the magnetic flux density of the transmitter/receiver coil of an inductive wireless power transfer system. The magnetic flux density of a transmitter was measured using a small, 3-axis search coil. The search coil was positioned in the 3D space above the transmitter coil using a 3D positioning mechanism and used to measure the magnetic flux density at a specific point. The data was then sent to a computer application to visualize the magnetic flux density. The measured magnetic field could be used in combination with electromagnetic field solvers to design and optimize transmitter coils for inductive wireless power transfer systems.

## 1. Introduction

Over the last decade, interest in wireless power transfer has grown. The main contributing factor to this was increased demand for wireless charging of electric vehicles and portable electronics [1,2,3]. The most frequently used method for wireless power transfer is inductive wireless power transfer, or IPT. It has several advantages over other wireless power transfer methods, including safety, a wide power transfer range, scalability, and high efficiency at short and medium ranges. The power range of an IPT is usually between a few watts and several kilowatts, depending on the application. 

The most important parts of an IPT system are the transmitter and receiver coils, which are separated by an air gap that can range from a few millimeters to a few decimeters. The transmitter coil generates an alternating magnetic field that induces a voltage in the receiver coil and supplies power to the load. In a wireless charging application, the load is usually a battery. The most common type of coil used in IPT is a planar spiral coil with a circular, quadrature, or square footprint. It can be used in different applications, from mobile phones to electric car chargers. The dimensions of the planar spiral coil are usually determined by the application constraint, such as the available area, the air gap between the coils, and the thickness of the wire. The inductance of the coil is determined by the maximum transmitted power. The number of turns is determined by the inductance of the coil. The coil can be wound with high-frequency litz wire or printed on a circuit board. In high-frequency, high-power applications, the litz wire is necessary to reduce the AC resistance of the coil, and thus reduce the thermal losses of the IPT system. 

To better shape the radiated magnetic field, and to prevent organic and metallic foreign matter from interacting with the magnetic field, ferrite material is placed under the transmitter and on the receiver coil. Ferrite material ranges in shape from whole ferrite plates or blocks to ferrite rods. Ferrite plates are generally used in coils with smaller areas, while ferrite rods are used in larger coil areas. Ferrite rods have poorer shielding properties than ferrite plates but are less expensive and lower in weight than large ferrite plates. 

The shape and direction of the magnetic field radiated by the transmitter coil is determined by the shape of the coil, the number of turns, the diameter of the wire, and other physical parameters [4,5,6,7,8,9]. The strength of the transmitter coil field is determined by the physical parameters of the coil and the primary excitation current. The calculation of the magnetic field and the optimization of the coil structure are usually preformed with an EM field simulation software, such as Ansys Maxwell or COMSOL Multiphysics. The coil parameters can be optimized numerically and then used in the fabrication process of the IPT coils. The numerical calculations are demanding on time and computer resources. The practical measurements of magnetic flux density are not very common due to their inconvenience. In practical tests, the magnetic field is usually measured at specific, discrete points [10,11,12], to verify that the EM radiation emitted by the IPT system complies with safety guidelines and regulations [13,14].

This study aimed at presenting a measurement platform for automated magnetic flux density measurement in the space around the transmitter coil. The measurements were based on a search coil magnetometer mounted onto a 3D positioning mechanism. In the proposed platform, the positioning mechanism moves the search coil around the transmitter coil, and measures the field radiated from the transmitter coil. The system scans the magnetic field around the coil and records the data for visualization. The custom developed app communicates with the 3D positioning mechanism and the search coil measurement circuit. The app is used to determine the scan area, as well as for receiving, recalculating, and logging data. Data are visualized using a 3D magnetic flux density diagram, similar to the graphical representation of magnetic flux density in the EM simulation software. The measurement data can be used to test and develop new IPT transmitter coil topologies and to test the fabricated IPT transmitter coils. This could be especially useful if the fabricated coil were to have a more complex structure, which could not be easily modelled in the EM simulation software. The automated measurement of the magnetic field could also help to test the integration of the IPT transmitter coil in systems, such as mobile devices and laptops. An additional advantage of the measurement platform is that it could serve as a replacement for time-consuming computer simulations that require expensive programs and computer hardware. 

The magnetic field measurement system was based on the search coil magnetometer, which works on the principle of electromagnetic induction. The measurement system enables flexibility by choosing sense coils with different radii and number of turns, depending on the required resolution and magnetic field strength. In the case of transmitter coils with a smaller radius, the resolution of the magnetic field measurement should be larger. Likewise, in the case of larger transmitter coils, the resolution of the magnetic field measurement should be smaller. The search coil radius also impacts the minimum vertical *z* distance between the transmitter coil and the measurement search coil. The 3D search coil enables the measurement of all three magnetic flux density components in the point in space. On the other hand, if a sensor were composed of three sensors based on the hall effect, additional corrections to the measurements would be required in order to compensate for the position offset of the hall sensors. The measurement system was also designed with the replicability of the 3D search coil in mind. New 3D search coils are easy to fabricate; thus, measurements can be adapted to specific requirements. On the other hand, the development and fabrication of MEMS sensors require special manufacturing equipment and processes.

The paper is organized as follows. Section 2 described the magnetic flux measurement platform and was divided into three main parts. In the first part, we described the transmitter part of the IPT system, which drives the transmitter coil representing the device under test. In the second part of the section, we described the 3D positioning mechanism with mounted search coil and transmitter coil. The third part focused on the measurement circuit used to measure the voltage induced in the 3D search coil. In Section 3, we described the theory behind the search coil magnetometer. Section 4 contains the results of measuring the magnetic flux density of two different coil topologies. The measured results were compared with results obtained from the EM field simulation software. In Section 5, a discussion of the measured and simulated results was presented. Lastly, Section 6 served as a conclusion of the measurement circuit presented in this paper.

## 2. Measurement Platform Description

A measurement platform was fabricated to measure the magnetic flux density of the transmitter coil under test in the 3D space. The proposed magnetic flux density measurement platform consisted of three interconnected systems:

- A transmitter coil driving circuit;
- A search coil with the positioning mechanism;
- A search coil voltage measurement circuit.

The measurement platform was designed to work as follows: first, the transmitter coil driving circuit is connected to the transmitter coil and drives the coil with a sinusoidal input current. Then, the magnetic field generated by the transmitter coil is then picked up by a 3D search coil, which can be positioned in *x*, *y*, and *z* directions in the scan area around the coil using the 3D positioning mechanism. The 3D search coil is connected to a 3-channel high impedance voltage measurement circuit, which measures the amplitude of the induced voltage and transmits the value to the PC. The PC logs the data and controls the position of the search coil. The voltage data are converted into a *.csv file. The transmitter coil driving circuit, the transmitter coils, and the search coil were designed with replaceability in mind in case different coil or converter topologies were used.

**Figure 1:**
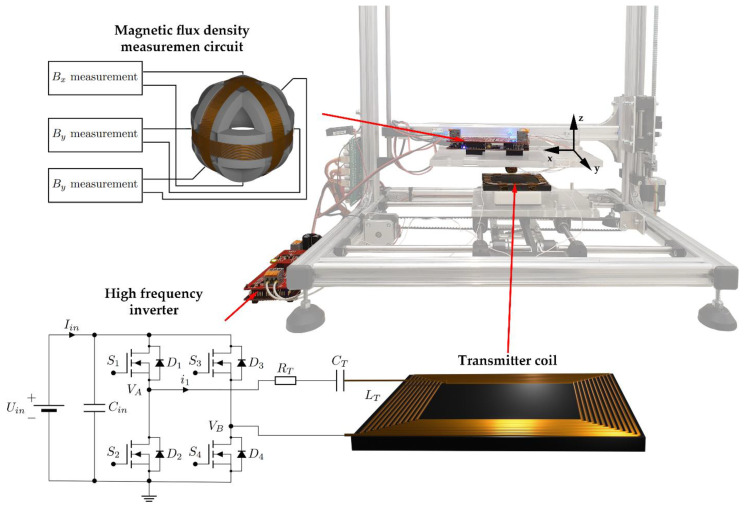
Magnetic flux density measurement platform.

The fabricated platform is presented in Figure 1. The main parts of the system included a high-frequency inverter, the transmitter coil and 3D search coil, and the voltage measurement system. The parts were mounted on the 3D positioning mechanism. The platform was connected to the PC.

### 2.1. Transmitter Coil Driving Circuit

A classic full-bridge high-frequency inverter was used to drive the transmission coil. The inverter converted the input DC voltage to a high-frequency AC voltage, which was used to drive the transmitter coil. To drive the transmitter coil with a sinusoidal current, the coil must be connected to a resonant compensation network. The main purpose of the compensation network was to compensate for the reactive power of the system’s IPT system. A compensation network usually consists of passive components, such as capacitors and inductors. In the system shown in Figure 1, the series (S) compensation scheme was used on the transmitter side. The transmitter coil was connected in series to a compensation capacitor with value *C_T_*. The compensation capacitor was selected based on the resonant frequency of the IPT system. The resonant frequency of the system (inductor *L_T_*, capacitor *C_T_*, and coil resistance *R_T_*) was adjusted to the high-frequency inverter switching frequency by designing the capacitor *C_T_* as follows:

(1) $$C_{T}={\frac{1}{{\left(2\pi f_{s}\right)}^{2}L}}_{T}$$

where *f_s_* is the switching frequency of the high-frequency inverter. The circuit of the inverter, compensation circuit, and transmitter coil are presented in Figure 1. The inverter consisted of a DC voltage source with voltage *U_in_* and four MOSFET transistors in full-bridge topology. The inverter was connected to the compensation capacitor *C_T_* and transmitter coil with inductance *L_T_*. The DC resistance of the coil and wires was presented with resistor *R_T_*.

**Figure 2:**
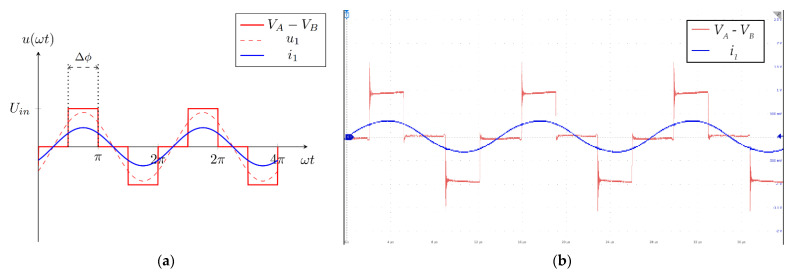
Voltage and current waveforms at the input of the resonant circuit: (**a**) theoretical waveforms; (**b**) waveforms measured on the experimental system.

The current and voltage at the input of the resonant IPT circuit are presented in Figure 2a,b. Figure 2a presents the theoretical waveforms. Each leg of the full-bridge inverter generated a square voltage with a 50% duty cycle. To control the resonator current *i*_1_, a phase shift was introduced between the half-bridge legs. The phase shift angle could be between 0 and π. In the figure, input voltage *V_A_* − *V_B_* is marked with a red line, while the main harmonic component that drives the current through the circuit is marked with the dashed red line *u*_1_. The resonant circuit served as a bandpass filter. The current through the resonant circuit *i*_1_ is marked with the blue line. Figure 2b presents the waveforms, measured on a real system. The red line represents the voltage at the input of the resonant circuit, and the blue line represents the sinusoidal current at the input of the resonant circuit.

The first harmonic component of the output voltage of the inverter was calculated from inverter input voltage *U_in_* and the phase-shift angle between the half-bridge legs of the inverter [2].

(2) $$u_{1}=\left(\frac{4U_{in}}{\pi }\sin\left(\frac{\phi }{2}\right)\right)\sin\left(\omega_{s}t\right)=U_{1}\sin\left(\omega_{s}t\right)$$

The series compensated transmitter resonator circuit can be described with the voltage equation:

(3) $$U_{1}=R_{1}I_{1}+U_{LT}+U_{CT}-U_{M}$$

where *U*_1_ is the amplitude of the first harmonic component of the inverter output voltage. *I*_1_ is the amplitude of the resonator current. *U_LT_* is the amplitude of the inductor voltage, and *U_CT_* is the amplitude of the capacitor voltage. The voltage *U_M_* was induced due to the currents in the 3D search coil. Because the search coil was connected to the high impedance measurement circuit, the current through each of its windings was very small. Therefore, the voltage *U_M_* induced by the search coil was almost zero and could be neglected. The *U_M_* stayed at zero regardless of the position of the search coil in the space.

By replacing the capacitor voltage and the inductor voltage with primary current and impedances, Equation (3) now takes form as:

(4) $$U_{1}=\left(R_{T}+j\omega L_{T}-j\frac{1}{\omega C_{T}}\right)I_{1}$$

where ω is the angular frequency of the high-frequency inverter. The Equation (4) was derived for the harmonically excited transmitter resonant circuit. The input impedance of the circuit can be expressed from (4) as:

(5) $$Z_{in}=\frac{U_{1}}{I_{1}}=R_{T}+j\omega L_{T}-j\frac{1}{\omega C_{T}}$$

In the case of resonance, the impedance of the capacitor and transmitter coil are the same and can be described with the condition:

(6) $$j\omega L_{T}-j\frac{1}{\omega C_{T}}=0$$

Under the condition of resonance, the resistance of the transmitter circuit is defined only by the resistance of the circuit:

(7) $$Z_{in}=R_{T}$$

If the transmitter coil does not transfer power to the receiver coil, the input current is only limited by the resistance of the primary side. This includes wire resistance on the resistance of the transistors, resistance of the compensating capacitor, and, finally, the resistance of the transmitter coil. In the resonance, the current through the unloaded transmitter coil is relatively high due to the low resonator resistance.

### 2.2. Fabricated Search Coils and Positioning Mechanism

The mechanical part of the magnetic flux density measurement platform consisted of a 3D positioning mechanism and a 3D search coil magnetometer. The 3D positioning mechanism was fabricated from an open-source 3D printer, Velleman K8200. The 3D printer was stripped of its printing nozzle and heated bed and accompanying thermistors. The metallic positioning platforms were replaced with platforms fabricated from a 1 cm thick acrylic plate. This was a necessity because the magnetic field from the IPT coil could interact with metal objects in the nearby space, which could alter the magnetic flux density and would present additional losses.

The stepper motors of the 3D printer mechanisms were controlled with a driver board based on Arduino UNO. The driver board was connected to the computer with a USB cable. The 3D printer driver communicated with the PC using G-code commands sent via a virtual serial COM port. The commands, important for the interaction between the PC and 3D positioning platforms, were homing and movement to an absolute or relative position in the 3D space.

**Figure 3:**
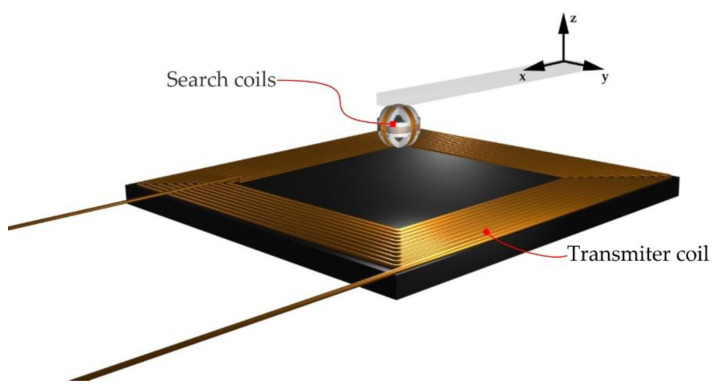
Transmitter coil and 3D search coil.

The 3D search coil was mounted on the top platform of the 3D positioning mechanism. The base of the 3D coil was fabricated from plastic using the 3D printer. Each of the single-axis 3D coils had the same dimensions and the number of turns. The radius of each coil was 5.5 mm and number of turns N was 30. The 3D search coil and the transmitter coil positioned in the 3D space are presented in Figure 3. The bottom platform with the transmitter coil moves in both *x* and *y* directions, and the top platform with the 3D search coil moves in the *z*-direction.

The parameters of the fabricated search coil used by the magnetometer are presented in Table 1. The self-inductance of the coils was not required as the system did not calculate mutual inductance. Only the number of turns and surface of each coil were required to calculate magnetic flux density from the induced voltage. The diameter of the search coil was 11 mm. This resulted in a small surface area of the coil itself, allowing more accurate measurement of the magnetic flux density at a point in space. The number of turns was chosen so that even a small magnetic flux density could induce a sufficiently large voltage on the search coil.

### 2.3. Fabricated Search Coils and Positioning Mechanism

**Figure 4:**
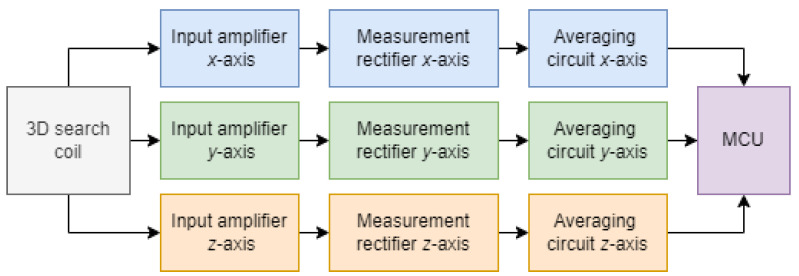
Block scheme of the measurement system.

If the 3D search coil was placed in the varying magnetic field, the voltage was induced across the terminals. This voltage was then measured and converted to a digital value with the analogue–digital converter of the microprocessor. The induced voltage was sinusoidal, due to sinusoidal magnetic flux density. The measurement circuit converted the open circuit AC voltage into a DC voltage, which was then sampled using an AD converter. The value of the DC voltage was then used to calculate the amplitude of the input voltage, and, later, the magnetic flux density, based on the coil’s parameters. Each of the three single-axis search coils required its own measurement circuit. A block scheme of the measurement system is presented in Figure 4.

**Figure 5:**
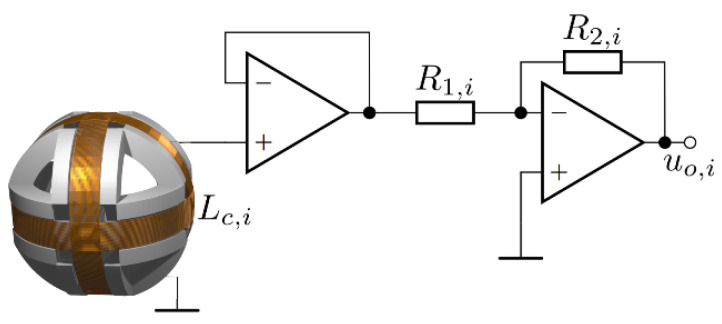
Input buffer and amplifier.

The measurement circuit consisted of three parts: an inverting amplifier, a precision rectifier, and an averaging circuit with operational amplifiers. The inverting amplifier, together with an input buffer circuit, is presented in Figure 5. The single-axis search coil was denoted with inductance *L_c,i_*, where *i* can be *x*, *y*, or *z*. One terminal of the coil was connected to the ground and the other terminal was connected to the input of the measurement circuit.

The buffer amplifier served as a high impedance load. The resistance of the coil could be neglected. Because the input resistance was so large, the input current was so small that it could also be neglected. Therefore, the measurement search coil did not induce a voltage in the transmitter coil circuit. The second part of the input amplifier circuit was the inverting amplifier. The gain of the amplifying part of the circuit is denoted with *G_i,_* and is defined with the values of the resistors *R*_1,*i*_, and *R*_2,*i*_. The gain *G_i_* can be expressed with:

(8) $$G_{i}=-\frac{R_{2,i}}{R_{1,i}}$$

**Figure 6:**
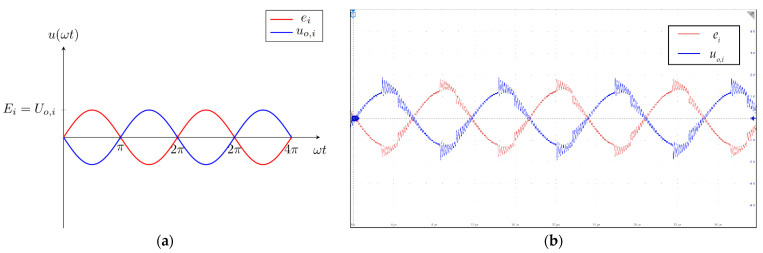
Input and output voltages of the first stage of the measurement circuit: (**a**) theoretical waveforms; (**b**) waveforms measured on the measurement system.

The measurement circuit had variable gain, to enable the measurements of different voltage ranges and, therefore, fields of different strengths. In the case of this paper, the gain was set to *G_i_* = 1 with two 10 kΩ resistors in all three measurement circuits. The voltage induced on the search coils was sufficiently high and did not require additional amplification. In the case that coils with fewer turns would be used instead, the unity gain could be appropriately set a higher value. The input and the output voltages are presented in Figure 6a,b. In Figure 6a, the red line is the induced voltage, and the blue line is the amplified voltage of the first stage. The output of the first stage was connected to the input of the second stage. In Figure 6b, the red signal is the input voltage induced in the search coil, and the blue line is the voltage at the output of the inverting amplifier.

**Figure 7:**
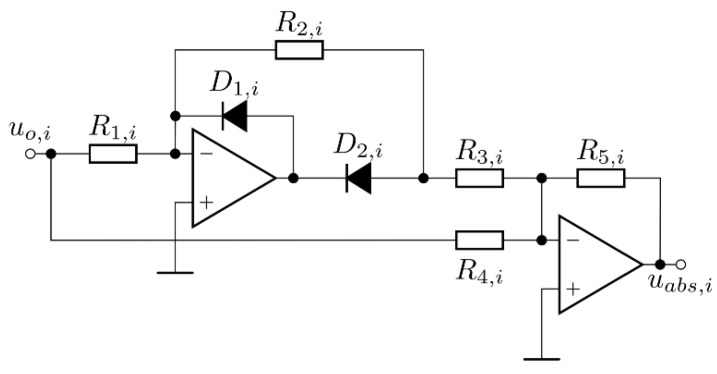
Measurement rectifier circuit.

The second part of the measurement circuit was a precision full wave-rectifier, which served to rectify the amplified input signal. It consisted of two amplifiers, fast signal diodes, and resistors. The full-wave rectifier is presented in Figure 7. The operational amplifier compensated for the voltage drop across the diode. The precision rectifier consisted of two stages: a precision half-wave rectifier and an addition circuit.

The gain of the precision half-wave rectifier with a gain was defined by *R*_1,*i*_ and R_2,*i*_. The second stage was the addition circuit, which added the original input signal to the output of the first stage. The gain of the addition circuit was determined with *R*_3,*i*,_ *R*_5,*i*_, and *R*_4,*i*_. In the case of the measurement circuit, the gain of the measurement rectifier was 1. The values of the resistors required for unity gain rectification can be calculated using:

(9) $$\begin{array}{l} R=R_{1,i}=R_{3,i}=R_{4,i}\\ R_{2,i}=R_{5,i}=2R \end{array}$$

**Figure 8:**
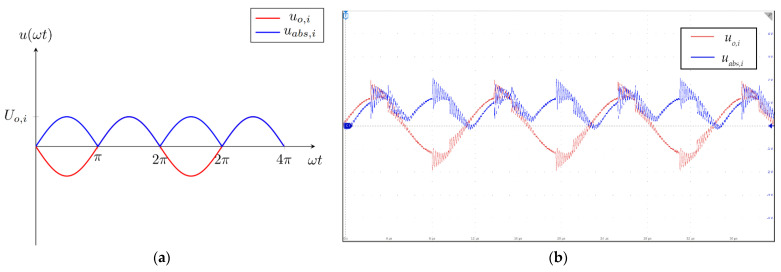
Input and output voltages of the second stage of the measurement circuit: (**a**) theoretical waveforms; (**b**) waveforms measured on the measurement system.

The input and the output voltages are presented in Figure 8a,b. In Figure 8a, the red line represents the voltage at the output of the inverting amplifier and the blue line represents the rectified voltage. The output of the second stage was connected to the input of the third stage. In Figure 8b, the red signal is the output voltage of the inverting amplifier and the blue line is the rectified voltage. Both signals measured on the amplifier circuit exhibited oscillatory behavior. The cause of the damped oscillations was the self-resonance of the measurement coil. The transmitter coil was driven using square voltage, which also had spikes due to the self-resonance of the transmitter coil. Those voltage spikes caused the impulse response in the measurement coils, which resulted in the oscillations present in measured voltages.

**Figure 9:**
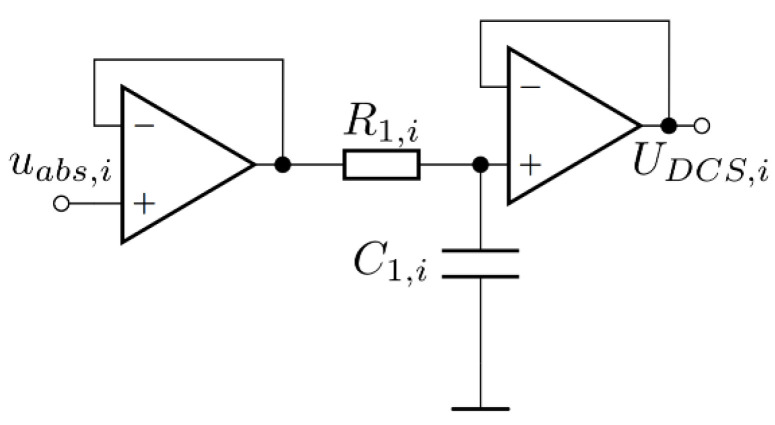
Measurement averaging circuit.

The third and last part of the measurement circuit was the averaging circuit. The purpose of this circuit was to convert the rectified AC voltage to DC voltage. The output of the circuit was the average value of the input voltage. The averaging circuit could also be replaced with a peak detector circuit. The circuit of the third and last stage is presented in Figure 9. It consisted of an input buffer amplifier, a low-pass RC filter, and an output buffer amplifier. The input voltage was rectified voltage *u_abs,i_*_,_ and the output voltage was the DC representation of the rectified AC voltage *U_DC_*_,*i*_.

**Figure 10:**
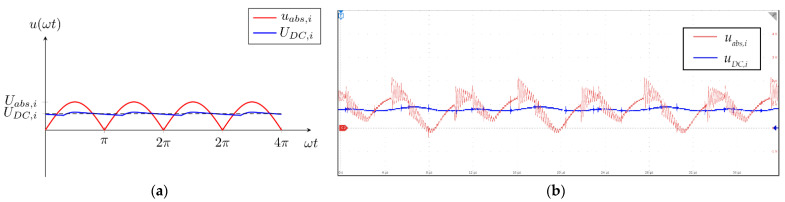
Input and output voltages of the third stage of the measurement circuit. (**a**) theoretical waveforms; (**b**) waveforms measured on the measurement system.

The input and output signals of the averaging circuit are presented in Figure 10a,b. In Figure 10a, the input rectified sinusoidal signal is marked with the red line and the output average value is marked with the blue line. In Figure 10b, the rectified voltage signal is marked with the red color and the DC average value is marked with the blue color. The oscillatory behavior of the induced and rectified AC voltage was filtered using the RC low-pass filter. As a result, the self-resonance of the measurement search coil did not impact the induced voltage measurement.

The circuit in Figure 9 averages the input waveform. The input of the circuit was the absolute sine waveform. Therefore, the average value *U_DC_*_,*i*_ of the voltage can be calculated using:

(10) $$U_{DC,i}=\frac{2}{\pi }U_{o,i}$$

The *U_DC,i_* was measured using the AD converter in the microcontroller. The peak value of the voltage *U_o_*_,*i*_ can be calculated using:

(11) $$U_{o,i}=\frac{\pi }{2}U_{DC,i}$$

The averaging window of the circuit (Figure 9) was defined using the filter resistor and capacitor. The time constant of the circuit was defined with *R*_1*,i*_ and *C*_1,*i*_:

(12) $$\tau_{avg}=R_{1,i}C_{1,i}$$

The accurate average value of the input voltage could be measured after a duration of five time constants. The value of the voltage was measured using the AD converter on the microprocessor. Because the measurement circuit was used in discrete measurement intervals, the microprocessor could sample the voltage multiple times and then calculate the average value:

(13) $$U_{DC,i}=\frac{\sum_{n=0}^{N}{\left[U_{DC,i}\right]}_{n}}{N}$$

### 2.4. Bandwidth Evaluation of the First Stage of Measurement System

When measuring magnetic flux density, the bandwidth of the measuring circuit must also be considered, especially the bandwidth of the operational amplifier (OA) system that presented in Figure 5. The input circuit included a buffer amplifier for impedance conditioning and an inverting amplifier. The frequency characteristics can be calculated as presented in [15].

The open-loop gain *A*(*j*ω) of the operational amplifier used in the measurement circuit was defined as:

(14) $$A\left(j\omega \right)=\frac{A_{o}}{1+\frac{j\omega }{\omega_{t}/A_{o}}}$$

where *A_o_* is the open-loop gain of the buffer amplifier at the frequency ω = 0 rad/s, and ω*_t_* is the cross-over frequency when the gain of the open-loop is 0 dB. When the resistances *R*_1,*i*_ and *R*_2,*i*_ are included in the OA system, the closed-loop gain can be calculated for the non-inverting OA system

(15) $$G_{1,i}\left(j\omega \right)=\frac{1}{1+\frac{1}{A_{o}(j\omega )}}$$

and for the inverting system (14):

(16) $$G_{1,i}\left(j\omega \right)=-\frac{R_{2,i}}{R_{1,i}}\frac{1}{1+\left(1+\frac{R_{2,i}}{R_{1,i}}\right)\frac{1}{A_{o}\left(j\omega \right)}}$$

After a short manipulation with (14) and (15) the gain for the non-inverting amplifier system was obtained:

(17) $$G_{1,i}\left(j\omega \right)=\frac{1}{1+\frac{j\omega }{\omega_{t}}}$$

The buffer amplifier with unity gain had the characteristics of the low-pass filter with the cutoff frequency at ω*_t_*, and after manipulation with (14) and (16), it was obtained:

(18) $$G_{2,i}\left(j\omega \right)=\frac{G_{i}}{1+\frac{j\omega }{\omega_{b,i}}}$$

where *G_i_* = *R*_2,*i*_/*R*_1,*i*_ and ω*_b_*_,*i*_ = ω*_t_*/(1 + *R*_2,*i*_/*R*_1,*i*_). The gain *G_i_* represents the closed-loop gain of the inverting amplifier at the frequency of zero. The transfer function of the first stage of the input measurement scheme could then be defined with the equation:

(19) $$G_{12,i}\left(j\omega \right)=\frac{G_{i}}{\left(1+\frac{j\omega }{\omega_{t}}\right)\left(1+\frac{j\omega }{\omega_{b,i}}\right)}$$

**Figure 11:**
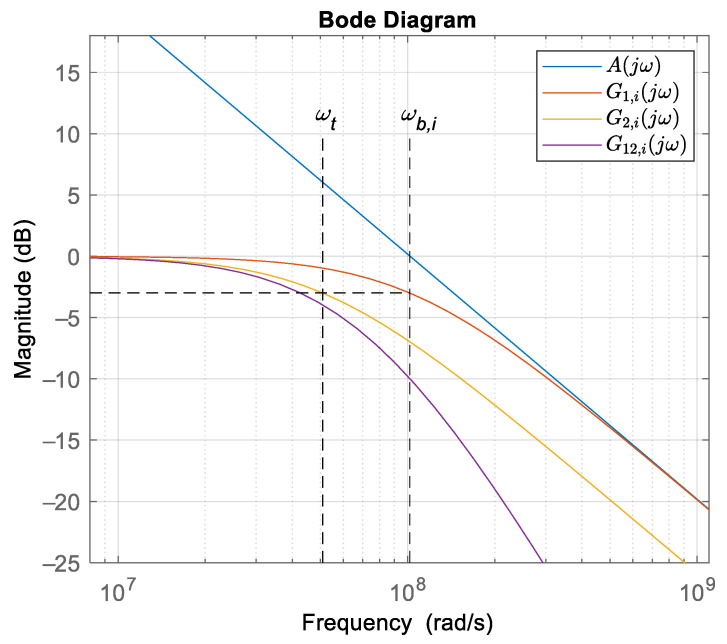
Bode plot of the measurement circuit.

A bode plot of the equations is presented in Figure 11. The blue line presents the characteristics of the amplifier gain, the red line is the characteristics of the buffer amplifier, the yellow line is the characteristics of the inverting amplifier with a gain of 1, and the purple line is the characteristics of the transfer function of the first stage of the input measurement scheme. The cut-off frequency of the buffer amplifier of the measurement circuit was equal to cross-over frequency (for chosen OA *f_t_* = 16.28 MHz) ω*_t_* = 1.02 × 10^8^, and the cut-off frequency of the second OA system (with resistors *R*_2,*i*_ and *R*_1,*i*_), ω*_b,i_* = ω*_t_*/2 = 5.11 × 10^7^ rad/s.

From (19) and Figure 11, it follows that the pass-band was defined when the transfer function *G_i_*(*j*ω) drop for −3 dB, which was equal to ω*_b,i_* and, according to the chosen measurement system frequency *f_s_* = 87 kHz (ω*_s_* = 5.47 × 10^5^ rad/s), the selected OA system satisfied the measurement requirements. Other OAs in the measurement chain had the same cross-over frequency.

Due to component tolerances, the frequency response of an inverting amplifier can be different from the ideal one. Thus, a sensitivity analysis was performed to predict the margin of error in the closed-loop gain due to the tolerances of the resistors in the circuit. The sensitivity function used in the analysis was defined as:

(20) $$S_{x}^{y}=\frac{\partial y}{\partial x}\frac{x}{y}$$

where *x* denotes the value of the component, or, in this case, closed-loop gain *G_i_* at frequency *ω_b_*_,*i*_ and *y* denotes the circuit parameter in interest, which is the closed-loop gain *G*_2,i_ in absolute form. The absolute value of (19) is:

(21) $$G_{A,i}=\left|G_{2,i}\left(j\omega \right)\right|=\frac{G_{i}}{\sqrt{1+{\left(\frac{j\omega }{\omega_{b,i}}\right)}^{2}}}$$

The gain *G_A_*_,*i*_ has sensitivities on two parameters, *G_i_* and ω*_b_*_,*i*_. The sensitivities were evaluated as follows:

(22) $$S_{G_{i}}^{G_{A,i}}=\frac{dG_{A,i}}{dG_{i}}\frac{G_{i}}{G_{A,i}}=1$$

(23) $$S_{\omega_{b}}^{G_{A,i}}=\frac{dG_{A,i}}{d\omega_{b}}\frac{\omega_{b}}{G_{A,i}}=\frac{\omega }{\omega_{b}^{2}+\omega^{2}}$$

The low-frequency gain of the inverting amplifier was dependent on resistances *R*_2,*i*_ and *R*_1,*i*_. If the resistors were chosen with ±1% tolerance, the gain *G_i_* had ±2% tolerance. From sensitivity Equation (22), the variation in closed-loop gain caused the following tolerance:

(24) $$\frac{dG_{A,i}}{G_{A_{i}}}=S_{G_{i}}^{G_{A,i}}\frac{dG_{i}}{G_{i}}=\pm 2\%$$

The sensitivity analysis for Equation (23) could be performed at two different frequencies of ω. The first frequency was at ω = 0 and the second was at ω = ω*_b_*_,*i*_:

(25) $${S_{\omega_{b,i}}^{G_{A,i}}|}_{\omega =0}=0$$

(26) $${S_{\omega_{b,i}}^{G_{A,i}}|}_{\omega =\omega_{b}}=\frac{1}{2}$$

The variation of the closed-loop gain can be expressed as:

(27) $${\frac{dG_{A,i}}{\omega_{b,i}}|}_{\omega =0}=S_{\omega_{b,i}}^{G_{A,i}}\frac{d\omega_{b,i}{}_{i}}{\omega_{b,i}}=0$$

(28) $${\frac{dG_{A,i}}{\omega_{b,i}}|}_{\omega =\omega_{b,i}}=S_{\omega_{b,i}}^{G_{A,i}}\frac{d\omega_{b,i}{}_{i}}{\omega_{b,i}}=\pm 0.95\%$$

As stated before, because of the variation in the feedback loop resistance, the closed-loop gain was also variable. From the sensitivity analyses (24), (27) and (28) it was concluded that the maximum change in the closed-loop gain was in the range of ±2%.

### 2.5. Measurement Application

**Figure 12:**
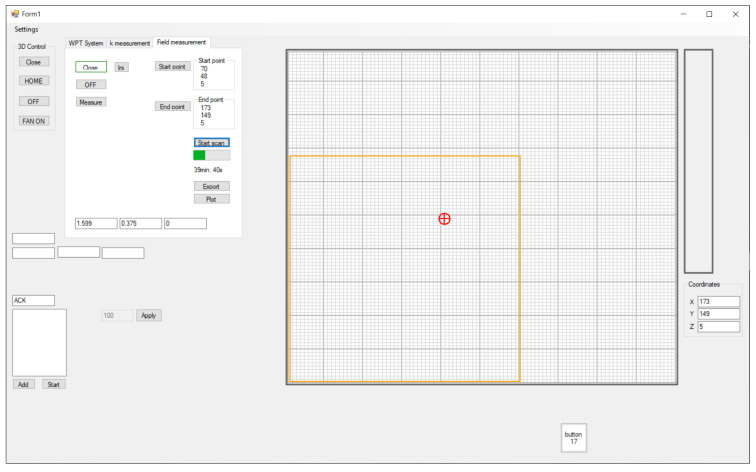
Control application for magnetic field measurement.

The computer application for measuring magnetic flux density was developed using the Microsoft Visual Studio using C#. The user interface of the application is presented in Figure 12. The interface included the measurement circuit control panel and the transmitter coil position grid. The main purpose of the application was the interaction between the 3D positioning mechanism and the search coil magnetometer. The graphic positioning grid interface was used for positioning the bottom platform with the transmitter coil in the *x* and *y*-axes and setting the vertical *z* distance between the 3D search coil and the transmitter coil. 

**Figure 13:**
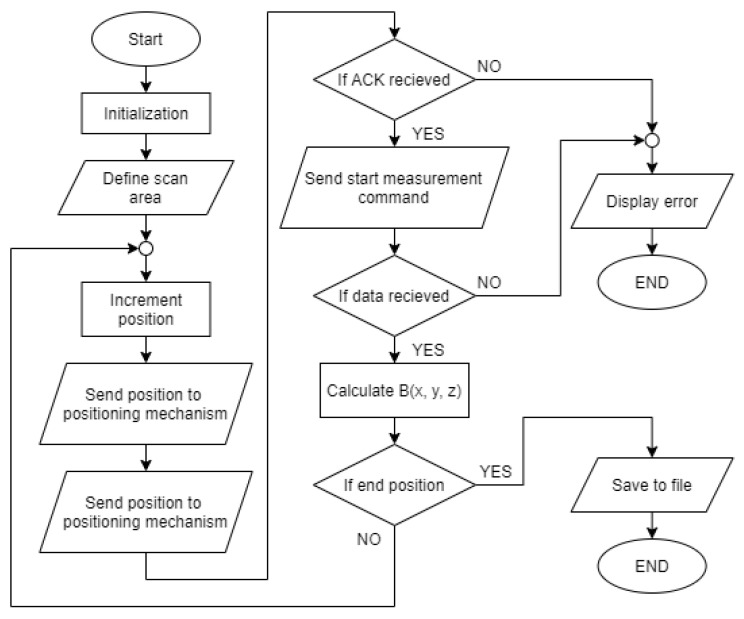
Measurement procedure flowchart.

To scan the magnetic field of the transmitter coil, the boundaries of the scan area must be defined using the start and the endpoints. The user can also determine the scanning step size. The smallest movement step in the *x*, *y*, or *z* direction was set at 1 mm. With the scan area defined, the measurement of magnetic flux density can start. The search coil moves incrementally within the designated area. At each point in the space, the application sends the command to the search coil magnetometer to perform the voltage measurement. Data are then transferred from the search coil magnetometer to the application. The magnetic flux density is then calculated and saved in the Random Access Memory (RAM). If the measurement data is transferred correctly, the coil can be moved into the next position. At the end of the measurement, data from the RAM can be exported as a .csv file for further analysis and visualization. The measurement procedure is described using the flowchart in Figure 13.

The app also included a 3D visualization function. The saved data, with extension .csv, can be imported and visualized in the 3D space. Each of the three components and the absolute value of the magnetic flux density can be represented with a magnetic flux density diagram.

## 3. Search Coil Magnetometer

**Figure 14:**
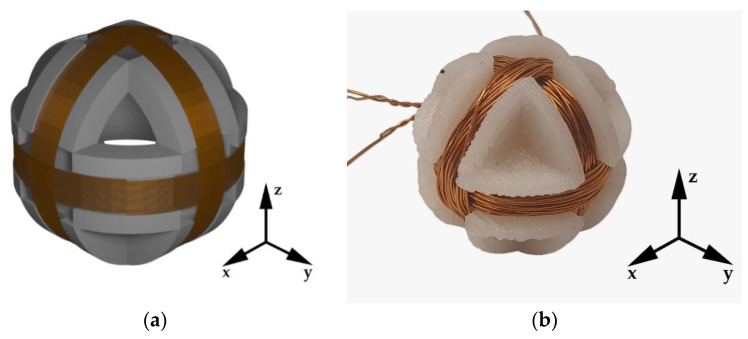
3D search coil: (**a**) 3D model; (**b**) Fabricated 3D search coil.

The main part of the search coil magnetometer was a 3D search coil without a ferromagnetic core. The purpose of the search coil was magnetic field measurement in the 3D space, i.e., in the *x*, *y,* and *z* directions. The 3D search coil consisted of three single axis search coils. The single axis search coils ere wound around the sphere and rotated 90° to each other. Each of the three coils was used to measure magnetic flux density along one specific axis. Because of the position and angle between the coils, the coupling coefficient between the coils was zero. This meant that the *x* component of magnetic flux density induced voltage only in the *x*-axis sense coil, i.e., it induced no voltage in the other two coils. The same was true for the *y* and *z* components of the magnetic flux density, which induced voltage only in the *y* and *z* single axis search coils, respectively. The modeled and fabricated 3D search coils are presented in Figure 14.

A wireless power transmitter coil, measured using the search coil magnetometer, usually generates magnetic flux in all three directions. Each component of an electric field induces a voltage in one of the search coils. The induced voltage is proportional to the magnetic flux density.

**Figure 15:**
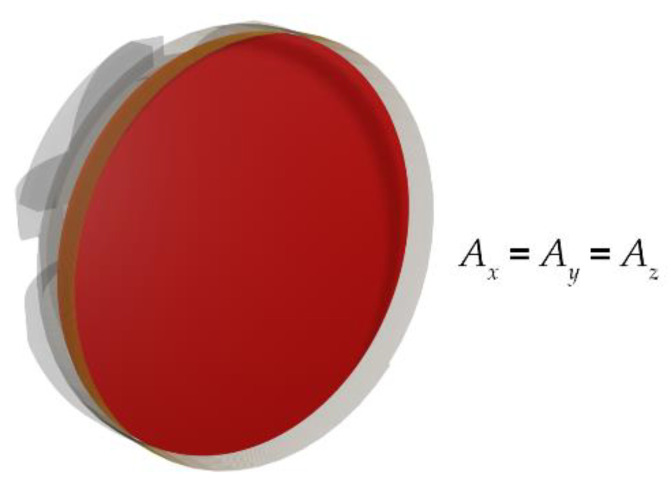
Cross-section area of the search coil magnetometer.

In this paper, the 3D search coil was used in connection with the transmitter side of a typical IPT system. The primary inverter side of the IPT system was used to drive the transmitter coil with a sinusoidal current. The excited transmitter coil generated a magnetic field. The shape of the magnetic field was defined by the geometric properties of the coil. The strength of the field was determined by the primary transmitter coil current. To measure the generated magnetic field, the 3D search coil was moved in the space around the transmitter coil. The voltages induced on the 3D search coils were measured in each point. The connection between magnetic flux and the induced voltage was described using Faraday’s induction law,

(29) $$\left[\begin{array}{c} e_{x}\left(t\right)\\ e_{y}\left(t\right)\\ e_{z}\left(t\right) \end{array}\right]=\left[\begin{array}{ccc} -N_{x} & 0 & 0\\ 0 & -N_{y} & 0\\ 0 & 0 & -N_{z} \end{array}\right]\left[\begin{array}{c} \frac{d\phi_{x}\left(t\right)}{dt}\\ \frac{d\phi_{y}\left(t\right)}{dt}\\ \frac{d\phi_{z}\left(t\right)}{dt} \end{array}\right]$$

where *e_x_*_,_ *e_y_* and *e_z_* are the voltage induced across the *x*, *y*, and *z* axis search coil, and *ϕ_x_*, *ϕ_y_*, and *ϕ_z_* are the magnetic flux in the *x*, *y*, and *z*-axes directions. The connection between the magnetic flux and magnetic flux density can be described with the equation

(30) $$\left[\begin{array}{c} e_{x}\left(t\right)\\ e_{y}\left(t\right)\\ e_{z}\left(t\right) \end{array}\right]=\left[\begin{array}{ccc} -N_{x}A_{x} & 0 & 0\\ 0 & -N_{y}A_{y} & 0\\ 0 & 0 & -N_{z}A_{z} \end{array}\right]\left[\begin{array}{c} \frac{db_{x}\left(t\right)}{dt}\\ \frac{db_{y}\left(t\right)}{dt}\\ \frac{db_{z}\left(t\right)}{dt} \end{array}\right]$$

where *N_x_*, *A_x_*, *N_y_*, *A_y_*, and *N_z_*, *A_z_* are the number of turns and cross-sectional area of the *x*, *y*, and *z* axis search coil, and *b_i_* is the varying magnetic flux density in the *x*, *y*, and *z*-axes. The cross-sectional area of the search coil magnetometer is presented in Figure 15. The cross-sectional area of all three search coils was the same.

By manipulating Equation (30), the magnetic flux density can be calculated by integrating the search coil voltage:

(31) $$\left[\begin{array}{c} b_{x}\left(t\right)\\ b_{y}\left(t\right)\\ b_{z}\left(t\right) \end{array}\right]=\left[\begin{array}{ccc} -\frac{1}{N_{x}A_{x}} & 0 & 0\\ 0 & -\frac{1}{N_{y}A_{y}} & 0\\ 0 & 0 & -\frac{1}{N_{z}A_{z}} \end{array}\right]\int \left[\begin{array}{c} e_{x}\left(t\right)\\ e_{y}\left(t\right)\\ e_{z}\left(t\right) \end{array}\right]dt$$

The magnetic field was generated by the sinusoidal excitation current. Therefore, the induced voltage was also sinusoidal, and could be described using:

(32) $$\left[\begin{array}{c} e_{x}\left(t\right)\\ e_{y}\left(t\right)\\ e_{z}\left(t\right) \end{array}\right]=\left[\begin{array}{c} {\hat{E}}_{x}\\ {\hat{E}}_{y}\\ {\hat{E}}_{z} \end{array}\right]\sin\left(\omega t\right)$$

where *Ê_x_*, *Ê_y_*, and *Ê_z_* are the amplitude of the voltage induced in the *x*, *y,* and *z* search coil, and ω is the angular frequency of the induced voltage. The integral of induced voltage could be calculated with the angular frequency of the inverter. Furthermore, the integral of the induced voltages (32) could be inserted in (31). The equation for calculating the magnetic flux density takes the form:

(33) $$\left[\begin{array}{c} b_{x}\left(t\right)\\ b_{y}\left(t\right)\\ b_{z}\left(t\right) \end{array}\right]=\left[\begin{array}{c} {\hat{B}}_{x}\\ {\hat{B}}_{y}\\ {\hat{B}}_{z} \end{array}\right]\cos\left(\omega t\right)=\frac{1}{\omega }\left[\begin{array}{ccc} \frac{1}{N_{x}A_{x}} & 0 & 0\\ 0 & \frac{1}{N_{y}A_{y}} & 0\\ 0 & 0 & \frac{1}{N_{z}A_{z}} \end{array}\right]\left[\begin{array}{c} {\hat{E}}_{x}\\ {\hat{E}}_{y}\\ {\hat{E}}_{z} \end{array}\right]\cos\left(\omega t\right)$$

At each point in the *x*, *y*, and *z* space, due to the change in magnetic flux density, the value of the induced voltage also changed. The amplitude of the magnetic flux density in the specific point in space can be calculated using:

(34) $$\vec{\hat{B}}=\left[\begin{array}{c} {\hat{B}}_{x}\left(x,y,z\right)\\ {\hat{B}}_{y}\left(x,y,z\right)\\ {\hat{B}}_{z}\left(x,y,z\right) \end{array}\right]=\frac{1}{\omega }\left[\begin{array}{ccc} \frac{1}{N_{x}A_{x}} & 0 & 0\\ 0 & \frac{1}{N_{y}A_{y}} & 0\\ 0 & 0 & \frac{1}{N_{z}A_{z}} \end{array}\right]\left[\begin{array}{c} {\hat{E}}_{x}\left(x,y,z\right)\\ {\hat{E}}_{y}\left(x,y,z\right)\\ {\hat{E}}_{z}\left(x,y,z\right) \end{array}\right]$$

The Equation (34) could then be used to calculate the magnetic flux density in three axes. To calculate the actual value of magnetic flux density in a specific point in the 3D space, the absolute value must be calculated from the measurements in all three axes:

(35) $$\left|\hat{B}\right|=\sqrt{{\hat{B}}_{x}{}^{2}+{\hat{B}}_{y}{}^{2}+{\hat{B}}_{z}{}^{2}}$$

The voltage induced in the search coil was measured with the previously described high impedance measurement circuit. The circuit transformed the AC voltage into DC voltage, which could be measured using the microcontroller. The measured DC voltage was used to calculate the amplitude of the magnetic flux density:

(36) $$\left[\begin{array}{c} {\hat{B}}_{x}\\ {\hat{B}}_{y}\\ {\hat{B}}_{z} \end{array}\right]=\frac{\pi }{2\omega }\left[\begin{array}{ccc} \frac{1}{N_{x}A_{x}} & 0 & 0\\ 0 & \frac{1}{N_{y}A_{y}} & 0\\ 0 & 0 & \frac{1}{N_{z}A_{z}} \end{array}\right]\left[\begin{array}{c} \frac{R_{2,x}}{R_{1,x}}U_{DC,x}\\ \frac{R_{2,y}}{R_{1,y}}U_{DC,y}\\ \frac{R_{2,z}}{R_{1,z}}U_{DC,z} \end{array}\right]$$

Because the amplitude of the induced voltage, measured during testing, was high enough, no additional amplification was required. The resistors *R*_2,*x*_, and *R*_1,*x*_ were set to the fixed value of 10 kΩ. Therefore, the gain of the measurement circuit was 1.

### 3.1. Bandwidth and Self-Resonance of the Search Coil Magnetometer

**Figure 16:**
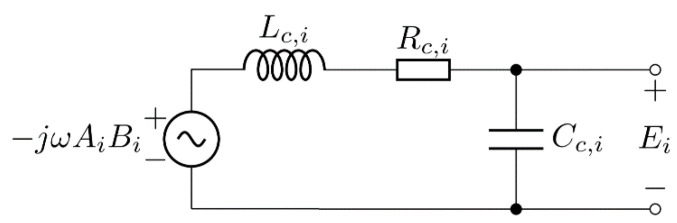
Equivalent circuit representation of the search coil.

The search coil of the magnetometer can be modeled using the AC voltage source, self-inductance *L_c_*_,*i*_, parasitic capacitance *C_c_*_,*i*_, which includes search coil capacitance and parasitic capacitance of the first amplifier stage, and DC resistance *R_c_*_,*i*_, where *i* = *x*, *y*, *z*. The circuit is presented in Figure 16. Induced voltage can be measured across the parasitic capacitance *C_c_*_,*i*_ using high impedance amplifier.

The transfer function of the search coil can be defined as:

(37) $$G_{c,i}\left(j\omega \right)=\frac{E_{i}\left(j\omega \right)}{B_{i}\left(j\omega \right)}=\frac{N_{i}A_{i}\omega }{R_{c,i}C_{c,i}+j\left(1-C_{c,i}L_{c,i}\omega^{2}\right)}$$

The resonant frequency of the coil can be described with:

(38) $$\omega_{r,i}=\frac{1}{\sqrt{L_{c,i}C_{c,i}}}$$

**Figure 17:**
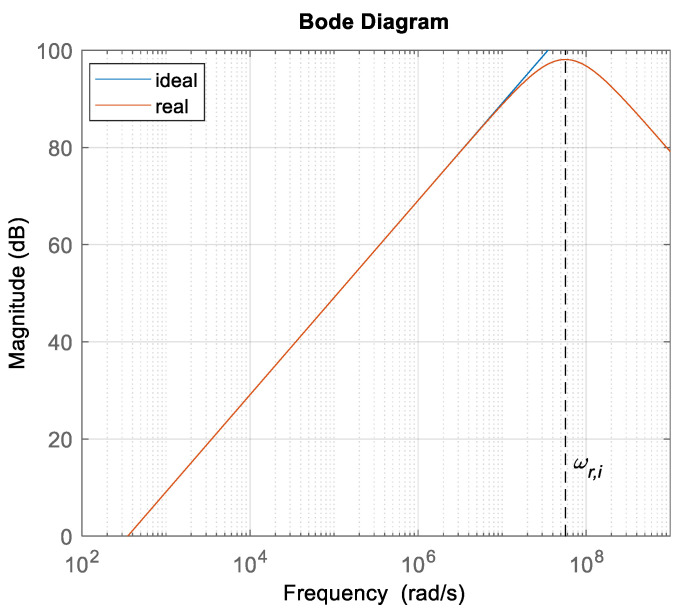
Bode plot of the search coil transfer function.

In the case of a 3D flux magnetometer, all coils have the same parameters. The self-inductance *L_c_*_,*i*_ was 17.6 μH, the parasitic capacitance *C_c_*_,*i*_ was 17.8 pF and the resistance of the wire was 1.4 Ω. Therefore, the self-resonant frequency of the single-axis search coil was 9 MHz (56.5 × 10^6^ rad/s). The bode plot of transfer function (37) is presented in Figure 17. The blue line represents the real transfer function with resonant frequency and the red line represents the ideal transfer function of the search coil. Under the resonant frequency, the ideal search coil behaved similarly to the nonideal search coil. The magnetic field measurements performed with the proposed system were under the self-resonant frequency of the coil. Therefore, the bandwidth of the magnetic field measurement circuit was limited by the precision rectifier and the RC filter.

### 3.2. Voltage Conversion Error of the Search Coil Magnetometer

As described in [16], the error of the magnetic field to voltage conversion can be expressed with noise equivalent magnetic induction (*NEMI*). *NEMI* is defined as the output voltage noise of the search coil magnetometer in relation to the transfer function of the search coil magnetometer, expressed in $T/\sqrt{L_{c,i}C_{c,i}}$. *NEMI* can be expressed with the equation:

(39) $$NEMI=\frac{v_{i,noise}}{G_{c,i}}$$

where *v_i_*_,*noise*_ is the variance of the noise, due to the noise at the input of the first amplifier stage and the thermal noise:

(40) $$v_{i,noise}=\sqrt{v_{A,i}{}^{2}+4kR_{C,i}T}$$

where *v_A_*_,*i*_ is the variance of the noise at the input of the amplifier, *k* is Boltzmann constant and *T* is the temperature in Kelvin. Using the transfer function (37), *NEMI* can be expressed with:

(41) $$NEMI=\frac{\sqrt{v_{A,i}{}^{2}+4kR_{C,i}T}}{\omega A_{i}S_{i}}\left(R_{c,i}C_{c,i}+j\left(1-C_{c,i}L_{c,i}\omega^{2}\right)\right)$$

Because the search coil magnetometer measures varying magnetic fields with frequency under the resonant frequency of the coil, the Equation (40) can be simplified, by excluding the resonant frequency due to the search coil capacitance and self-inductance:

(42) $$NEMI=\frac{\sqrt{v_{A,i}{}^{2}+4kR_{C,i}T}}{\omega N_{i}A_{i}}$$

Form (42) it wasconcluded that *NEMI* increased with temperature and resistance of the search coil and decreased with the number of turns, and in a larger coil cross-section. It also decreased with the increment of the frequency of the measured magnetic field.

## 4. Measurement Results

**Figure 18:**
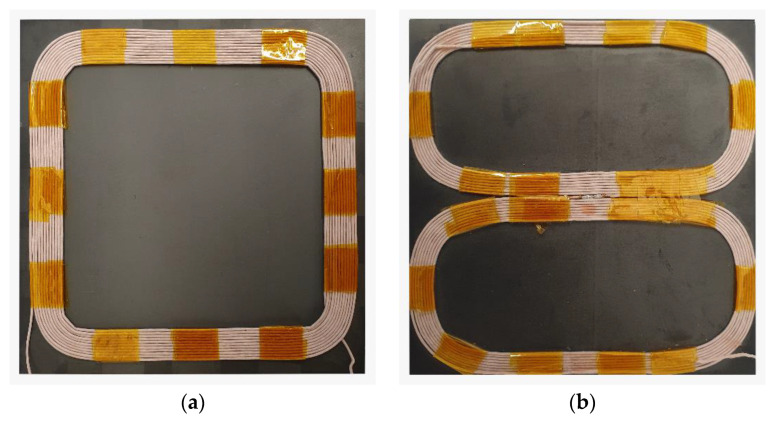
Transmitter coils under test: (**a**) Square planar coil; (**b**) DD coil.

To measure the magnetic flux density of the transmitter coil under the test in the 3D space, a measurement of the magnetic flux density was set up using the proposed system. The measurements were performed on two different coils: an ordinary, square planar coil on a square ferrite pad, and a directional DD coil on a square ferrite pad. Both coils are presented in Figure 18. Figure 18a presents the square planar coil with 10 turns, and Figure 18b presents the DD coil, which consisted of two rectangular coils with 9 turns. Both coils had nearly the same inductance.

The parameters of the used system are presented in Table 2, including the inductance of the square coil and the inductance of the DD coil, the transmitter coil excitation currents, input voltage of the inverter, and switching frequency of the phase-shifted PWM. The coils were designed to have similar inductance and footprints. Both coils were shielded with a 100 mm × 10 mm × 6 mm ferrite plate.

**Figure 19:**
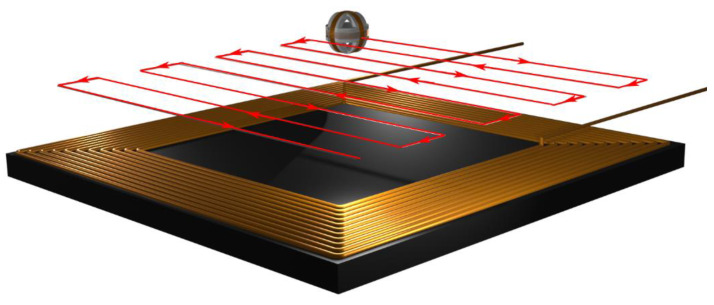
Scan path of the search coil in the plane *x-y*.

The measurements were performed in the *x-y* planes at different z distances. The search coil was moved on the *x-y* plane along with the user-defined path. The scan path of the search coil is presented in Figure 19 with red lines. The arrows indicate the direction of the movement. The search coil followed the path in discrete incremental movements. At the end of each incremental movement, the magnetic field was measured in the 3D space.

The extract of the experimental measurements is presented in Table 3 and Table 4 for the square planar coil and DD coil, respectively. The measurement was performed on the *x-y* plane at discrete distances. Three induced voltages were measured at each point on the plane. *Ê_x_* was the voltage induced in the *x*-axis search coil, *Ê_y_* was the voltage induced in the *y*-axis search coil, and *Ê_z_* was the voltage induced in the *z*-axis search coil. The induced voltage was proportional to the magnetic flux density. The relation between induced voltage and magnetic flux density is described with Equation (36).

**Figure 20:**
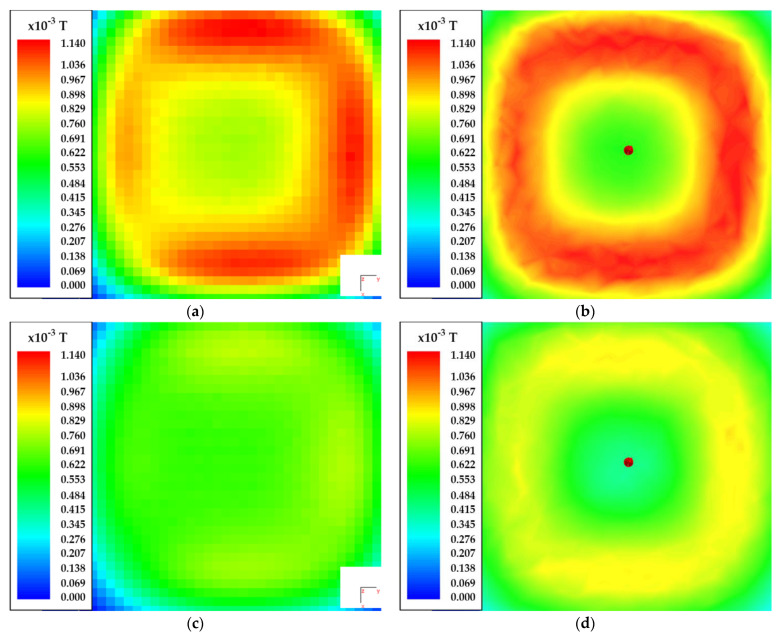

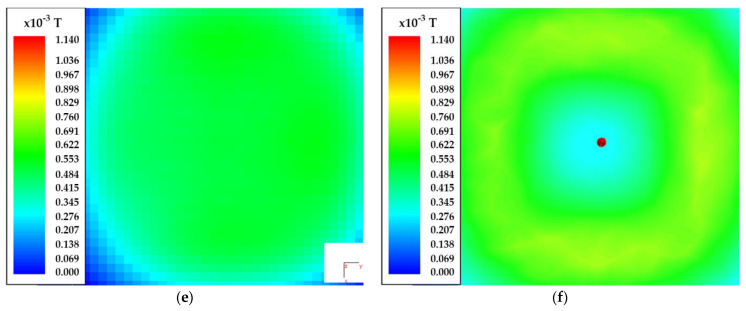
Magnetic flux density measurement of the square spiral transfer pad: (**a**) measured absolute value of magnetic flux density at *z* = 15 mm; (**b**) simulated absolute value of magnetic flux density at *z* = 15 mm; (**c**) measured absolute value of magnetic flux density at *z* = 20 mm; (**d**) simulated absolute value of magnetic flux density at *z* = 20 mm; (**e**) measured absolute value of magnetic flux density at *z* = 25mm; (**f**) simulated absolute value of magnetic flux density at *z* = 25 mm.

**Figure 21:**
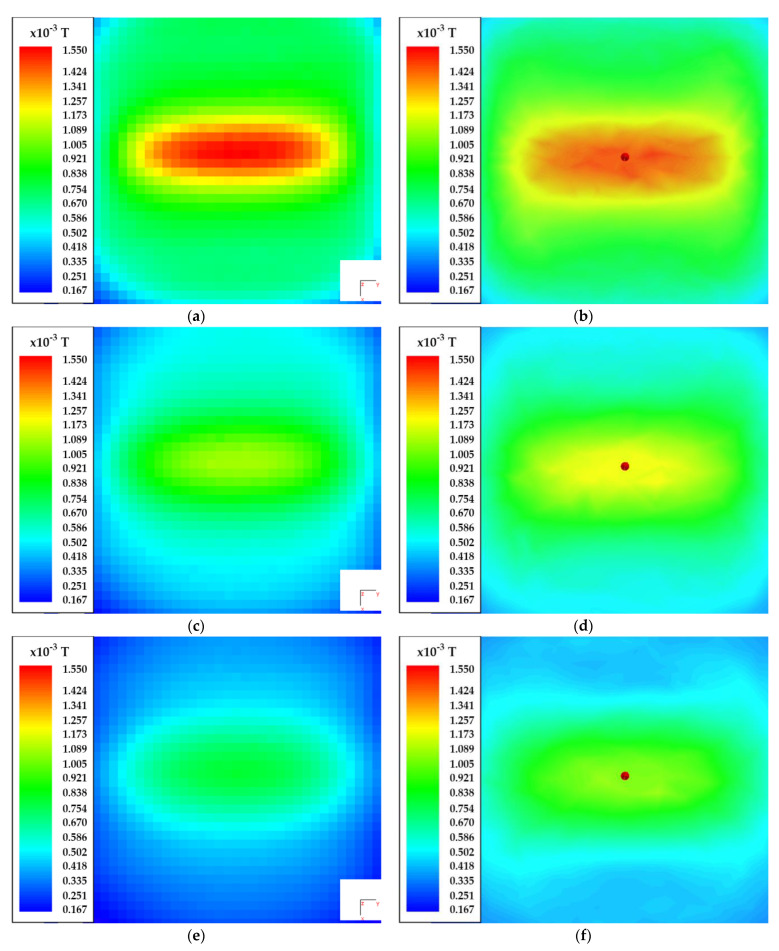
Magnetic flux density measurement of the DD transfer pad: (**a**) measured absolute value of magnetic flux density at *z* = 15 mm; (**b**) simulated absolute value of magnetic flux density at *z* = 15 mm; (**c**) measured absolute value of magnetic flux density at *z* = 20 mm; (**d**) simulated absolute value of magnetic flux density at *z* = 20 mm; (**e**) measured absolute value of magnetic flux density at *z* = 25 mm; (**f**) simulated absolute value of magnetic flux density at *z* = 25 mm.

The comparisons between the simulated and measured results are presented in Figure 20 and Figure 21, respectively. The diagrams present the absolute magnetic flux density in three different *x-y* planes, located at 15 mm, 20 mm, and 25 mm. The figures on the left side present the measured results and the figures on the right side present the simulated results. The results are presented using a color scale. The blue color defines the weakest magnetic flux density, and the red color defines the strongest magnetic field.

As described in the previous Section, the C# application was used to define the scan area and *z*-axis distance between the transfer coil and the 3D search coil. In both cases, the coils had the same, 10 cm × 10 cm footprint. The scan area was then defined as a 10 cm × 10 cm area around the centre of the coils. The measurements were performed at three z planes, at 15 mm, 20 mm, and 25 mm. The scan step on each *x-y* plane was set to the smallest value of 1 mm.

Figure 20 presents the measurement results of the spiral square coil, which radiated a nondirectional field. The current through the transmitter coil was set to 4 A. Figure 20a,b presents the measured and simulated magnetic flux density in the *x-y* plane at z = 15 mm. The biggest difference between the measured results and the simulation was at the center of the square coil. The measured magnetic field density was higher compared to the simulated value. Figure 20c,d presents the difference between the measured and simulated magnetic flux density at z = 20 mm. Similar to the previous case, the measured magnetic flux density at the center of the coil was higher than the simulated results. Finally, Figure 18 presents the measured and simulated magnetic flux density at z = 25 mm. Another notable difference in magnetic flux density was observed in the strength of the magnetic field in the center of the square coil.

The maximum value of magnetic flux density was above the wire at a distance of 15 mm. At the larger z distance, the magnetic flux density decreased, as can be seen in the measured and simulated figures. At all three distances, the measured magnetic flux density in the center of the coil was higher than the simulated values. When simulated, the maximum value of the magnetic flux density was above the conducting wire.

Figure 21 presents the measurement results of the DD coil, which radiated a directional field. The direction of the magnetic field was set along the *x*-axis. The current through the transmitter coil was set to 4 A. Figure 21a,b presents the measured and simulated magnetic flux density in the *x-y* plane at *z* = 15 mm. The biggest difference between the simulated and measured values of magnetic flux density was in the shape of the magnetic field. This difference was mainly due to the difference in the shape of the wound and 3D-modeled coils. Figure 21c,d presents the difference between the measured and simulated magnetic flux density at *z* = 20 mm. There was also some difference between the measured and simulated magnetic flux density. Finally, Figure 21e,f presents the measured and simulated magnetic flux density at *z* = 25 mm.

The maximum value of magnetic flux density was above the wire in the center of the coil at a distance of 15 mm. The maximum measured value was 1.55 mT. Compared to the magnetic flux density of the planar spiral coil, the magnetic flux density of DD coil was larger. In both cases, the coils were excited by a current with the same value. At greater distances, the magnetic flux density decreased, as can be seen in the measured and simulated figure data. Similar to the square planar coil, the measured magnetic flux density in the *z*-axis was more uniform than the simulated magnetic field, especially at greater z distances.

## 5. Discussion

The measured magnetic field could be used for wireless power transfer coil optimization. It could be used to visualize magnetic flux density in *x-y* planes at different *z*-axis distances. The measured magnetic field incuded *x*, *y* and, *z*-axis components. Each component could be displayed graphically. On the other hand, the simulation software was often only able to be used to present the absolute value of magnetic flux density. In the measurement results, only the absolute value of magnetic flux density was presented for the sake of comparison. The simulated magnetic flux density differed from the measured magnetic flux density. The largest differences were observed in the *z*-axis. The measured magnetic flux density in the *z*-axis was more uniform than the simulated value.

In IPT transmitter design, the most important direction of the field is the z-direction. This is because the receiver coil is usually oriented in the direction of the *z*-axis and in parallel to the transmitter coil; therefore, only the *z*-axis component of the magnetic field induces voltage in the receiver coil. If the receiver coil is not in parallel with transmitter coil and is rotated to it, the *x* and *y* components of the magnetic field also induce a voltage in the receiver coil and transfer power.

When designing wireless power transfer pads with better misalignment tolerance, the most important parameter is the magnetic flux density distribution in the *z*-axis. The transfer coils with the best magnetic flux density distributions are coils with a uniform directional magnetic field, such as DD coils.

The measurements were also performed with transmitter coils with a 12 mm thick ferrite plate. There was little to no difference observed between the measurement results with 6 mm thick ferrite. From a certain thickness on, ferrite did not significantly impact the magnetic field shape and strength.

A shortcoming of this system was that it was only able to evaluate performance and a field generated by a single transmitter coil. In this paper’s experimental configuration, it could not be used to measure the magnetic field between the transmitter and receiver coils during wireless power transfer. This was because the receiver coil could not be mounted on the current system. The measurement platform could, however, be expanded in the future, in order to allow the measurement of the magnetic flux during wireless power transfer.

## 6. Conclusions

This paper described the fabrication of a magnetic flux density measurement platform, designed for measurement and evaluation of different IPT transfer coil topologies. The measurement platform was designed based on a 3D search coil magnetometer and a 3D positioning mechanism. Due to Faraday’s Law of Induction, the varying magnetic field of the transmitter coil induced a voltage in the 3D search coil. The high impedance measurement circuit was used to measure and convert the induced AC voltage to DC voltage, which could then be measured using the AD converter and microcontroller. An application was also presented for interaction between a PC and the measurement platform. The measurement platform was used to evaluate the magnetic flux density of transmitter coils. The magnetic flux densities of two different transmitter coil structures were presented for comparison. The first was a planar square coil and the second was a DD coil. Additionally, the measurement results were compared to the results from EM simulation. The measurement platform could be used to evaluate and optimize wireless power transfer coils at the IPT design stage, together with EM simulation software. The platform would also be suitable for experimentation and development of new coil concepts. The platform was designed with flexibility in mind; for instance, different magnetometers could be used for magnetic field measurement. The search coil magnetometer could be replaced by a magnetometer, based on MEMS technology. In this paper, the search coil magnetometer enabled additional flexibility, which may not have been possible using different magnetic field measurement techniques.

## Figures and Tables

**Table 1:** 3D search coil parameters.

Parameter	Value
*x*-axis coil No. turns *N_x_*	30
*y*-axis coil No. turns *N_y_*	30
*z*-axis coil No. turns *N_z_*	30
*x*-axis coil surface A*_x_*	95.03 mm^2^
*y*-axis coil surface A*_y_*	95.03 mm^2^
*z*-axis coil surface A*_z_*	95.03 mm^2^

**Table 2:** Parameters of the test system.

Parameter	Value
Square planar coil inductance *L_sq_*	41.2 μH
DD coil inductance *L_dd_*	45 μH
Square coil current *I_sq_*	4 A
DD coil current *I_dd_*	4 A
Inverter input voltage *U_in_*	0 V–9 V
Inverter input current *I_in_*	0.5 A
Inverter frequency *f_s_*	87 kHz

**Table 3:** Magnetic flux density measurements of the square coil.

*x* (mm)	*y* (mm)	*z* (mm)	*Ê_x_* (V)	*Ê_y_* (V)	*Ê_z_* (V)	*B_x_* (T)	*B_y_* (T)	*B_z_* (T)	*B* (T)
0	0	15	0.192	0.257	0.235	1.23 × 10^−4^	1.65 × 10^−4^	1.51 × 10^−4^	2.55 × 10^−4^
0	5	15	0.304	0.397	0.239	1.95 × 10^−4^	2.55 × 10^−4^	1.53 × 10^−4^	3.56 × 10^−4^
0	10	15	0.413	0.575	0.193	2.65 × 10^−4^	3.69 × 10^−4^	1.24 × 10^−4^	4.71 × 10^−4^
0	15	15	0.464	0.779	0.11	2.98 × 10^−4^	5.00 × 10^−4^	7.06 × 10^−5^	5.86 × 10^−4^
0	20	15	0.447	0.983	0.026	2.87 × 10^−4^	6.31 × 10^−4^	1.67 × 10^−5^	6.93 × 10^−4^
0	25	15	0.37	1.15	0	2.37 × 10^−4^	7.38 × 10^−4^	0.00	7.75 × 10^−4^
0	30	15	0.261	1.27	0.012	1.67 × 10^−4^	8.15 × 10^−4^	7.70 × 10^−6^	8.32 × 10^−4^
0	35	15	0.165	1.334	0.025	1.06 × 10^−4^	8.56 × 10^−4^	1.60 × 10^−5^	8.63 × 10^−4^
0	40	15	0.087	1.398	0.027	5.58 × 10^−5^	8.97 × 10^−4^	1.73 × 10^−5^	8.99 × 10^−4^
0	45	15	0.027	1.424	0.025	1.73 × 10^−5^	9.14 × 10^−4^	1.60 × 10^−5^	9.14 × 10^−4^
0	50	15	0.001	1.43	0.02	6.42 × 10^−7^	9.18 × 10^−4^	1.28 × 10^−5^	9.18 × 10^−4^
0	55	15	0.008	1.433	0.016	5.13 × 10^−6^	9.19 × 10^−4^	1.03 × 10^−5^	9.20 × 10^−4^
0	60	15	0.05	1.411	0.012	3.21 × 10^−5^	9.05 × 10^−4^	7.70 × 10^−6^	9.06 × 10^−4^
0	65	15	0.109	1.393	0.009	6.99 × 10^−5^	8.94 × 10^−4^	5.77 × 10^−6^	8.97 × 10^−4^
0	70	15	0.181	1.339	0.004	1.16 × 10^−4^	8.59 × 10^−4^	2.57 × 10^−6^	8.67 × 10^−4^
0	75	15	0.272	1.263	0	1.75 × 10^−4^	8.10 × 10^−4^	0.00	8.29 × 10^−4^
0	0	15	0.192	0.257	0.235	1.23 × 10^−4^	1.65 × 10^−4^	1.51 × 10^−4^	2.55 × 10^−4^
…	…	…	…	…	…	…	…	…	…

**Table 4:** Magnetic flux density measurements of the DD coil.

*x* (mm)	*y* (mm)	*z* (mm)	*Ê_x_* (V)	*Ê_y_* (V)	*Ê_z_* (V)	*B_x_* (T)	*B_y_* (T)	*B_z_* (T)	*B* (T)
0	0	0	0.353	0.287	0.082	2.27 × 10^−4^	1.84 × 10^−4^	5.26 × 10^−5^	2.97 × 10^−4^
0	5	0	0.406	0.436	0.18	2.61 × 10^−4^	2.80 × 10^−4^	1.15 × 10^−4^	3.99 × 10^−4^
0	10	0	0.408	0.591	0.301	2.62 × 10^−4^	3.79 × 10^−4^	1.93 × 10^−4^	5.00 × 10^−4^
0	15	0	0.355	0.729	0.417	2.28 × 10^−4^	4.68 × 10^−4^	2.68 × 10^−4^	5.85 × 10^−4^
0	20	0	0.277	0.83	0.508	1.78 × 10^−4^	5.33 × 10^−4^	3.26 × 10^−4^	6.49 × 10^−4^
0	25	0	0.196	0.909	0.581	1.26 × 10^−4^	5.83 × 10^−4^	3.73 × 10^−4^	7.04 × 10^−4^
0	30	0	0.123	0.943	0.617	7.89 × 10^−5^	6.05 × 10^−4^	3.96 × 10^−4^	7.27 × 10^−4^
0	35	0	0.066	0.966	0.642	4.23 × 10^−5^	6.20 × 10^−4^	4.12 × 10^−4^	7.45 × 10^−4^
0	40	0	0.025	0.983	0.662	1.60 × 10^−5^	6.31 × 10^−4^	4.25 × 10^−4^	7.61 × 10^−4^
0	45	0	0.008	0.982	0.67	5.13 × 10^−6^	6.30 × 10^−4^	4.30 × 10^−4^	7.63 × 10^−4^
0	50	0	0.008	0.983	0.68	5.13 × 10^−6^	6.31 × 10^−4^	4.36 × 10^−4^	7.67 × 10^−4^
0	55	0	0.016	0.975	0.682	1.03 × 10^−5^	6.26 × 10^−4^	4.38 × 10^−4^	7.64 × 10^−4^
0	60	0	0.046	0.981	0.693	2.95 × 10^−5^	6.29 × 10^−4^	4.45 × 10^−4^	7.71 × 10^−4^
0	65	0	0.098	0.958	0.683	6.29 × 10^−5^	6.15 × 10^−4^	4.38 × 10^−4^	7.58 × 10^−4^
0	70	0	0.177	0.94	0.669	1.14 × 10^−4^	6.03 × 10^−4^	4.29 × 10^−4^	7.49 × 10^−4^
0	75	0	0.279	0.912	0.636	1.79 × 10^−4^	5.85 × 10^−4^	4.08 × 10^−4^	7.36 × 10^−4^
0	0	0	0.353	0.287	0.082	2.27 × 10^−4^	1.84 × 10^−4^	5.26 × 10^−5^	2.97 × 10^−4^
…	…	…	…	…	…	…	…	…	…

## Data Availability

Data are contained within the article at hand.
